# Human iPSC-Chimera Xenotransplantation and the Non-Identity Problem

**DOI:** 10.3390/jcm8010095

**Published:** 2019-01-15

**Authors:** Paula Casal, Andrew Williams

**Affiliations:** 1 Institució Catalana de Recerca i Estudis Avançats (ICREA), Pg. Lluís Companys 23, 08010 Barcelona, Spain; 2 Universitat Pompeu Fabra, Law Department, Trías Fargas 25, 08005 Barcelona, Spain

**Keywords:** chimeric pigs, chimpanzees, CRISPR, ethics, Non-Identity Problem, nonhuman person, xenotourism, xenotransplantation, zoonotic risks

## Abstract

Xenotransplantation is often deemed morally objectionable because of the costs it imposes on the organ donor and the risks it imposes on the recipient. For some, involving human–pig chimeras as donors makes the practice more objectionable or even abhorrent from the start. For others, by contrast, using such chimeras weakens recipient-based objections because it reduces the risk of organ rejection and malfunctioning, and cancels donor-based objections because the practice does not harm chimeras but instead gives them valuable lives they would not otherwise have. The paper examines and eventually rejects the latter defense. It also discusses the additional risks of chimeric xenotourism in countries with less demanding procedural guidelines and reflects on two very different futures for humanity that may emerge from supporting or rejecting chimeric xenotransplantation.

## 1. Introduction

Organ transplantation raises numerous ethical issues. Even transplanting kidneys from deceased human donors with their prior consent can raise some ethical concerns. For example, we may wonder about the conditions under which their consent is secured, or question a definition of death [1,2]; we may find the content and consequences of their consent objectionable, as when racist donors agree to transplants provided priority is given to members of their own race when distributing their organs [3]; or we may object to transplants of certain organs, such as wombs, because of the possible effects on third parties, such as the baby growing with immunosuppressants [4].

In cases of *xenotransplantation*, when the donor and the recipient belong to different species, further issues arise, regarding (i) the unavoidable harms to the *donor* and (ii) the possible harms to the *recipients*. When the donor is a chimeric pig, designed to contain a percentage of human genes and cells, xenotransplantation provokes two opposing sets of reactions [5].

For some, the use of human-pig chimeras strengthens both *recipient-based* and *donor-based* objections, rendering xenotransplantation more objectionable or even impermissible. Many feel an aversion to such a deep transgression of the natural order, for reasons that may be theological, ecological, or involve human dignity, animal rights or fear of scientists yet again producing undesirable, unforeseen, and irreversible consequences. Others have more specific concerns. For example, the US National Institutes of Health suspended funding of their chimeric xenotransplantation program in 2015 over concerns about introducing human DNA into a member of another species, and particularly doing so at such an early developmental stage. As Carrie Wolinetz, Salz Institute’s Associate Director for Science Policy, explains: ‘people were concerned about human cells populating the brain of the animal or the germline of the animal’ [6]. In the first case, the animal might acquire morally relevant human traits; in the second case, the animal might transmit human genes and traits to its offspring, for example, if accidentally released into the wild. In addition, gene editing is not allowed in humans because it is associated with off-target mutations that can cause cancer and other mutations and can be passed on to a patient’s descendants [7].

For a variety of reasons, then, some find the involvement of human-pig chimeras particularly problematic. Others experience the opposite reaction. They appeal to the fact that the human component of the donor will greatly reduce the probability of organ rejection, malfunction, and ill-fitting physiology, thereby weakening the recipient-based case against xenotransplantation. They also note that gene editing permits the removal of genes associated with disease. In addition, perfecting the technique of mixing the DNA of some species with that of others will eventually enable humans not only to edit out any genes in the animal donor that pose zoonotic risks for humans, but perhaps also enable them to import into human bodies the superior capacity of other species to deal with infection or disease. One example is the acquisition of crocodiles’ high resistance to infection or naked mole rats’ resistance to cancer and pain [8,9,10].

Finally, the use of purpose-made chimeras may also undermine the donor-based objections to xenotransplantation since chimeric donors would have never existed outside a xenotransplantation program, and thus owe their existence to it. If chimeras are well cared for until their moment of death, they will have lives worth living that they would not otherwise enjoy. Chimeras may, then, be seen not as the victims, but perhaps even beneficiaries of the program. This way, both donor-based objections and recipient-based objections, which appeal to the moral costs to the beneficiary of having harmed and then killed the donor, a creature that in some respects is a relative, are also canceled out, as the suitably humanized chimeras have valuable lives and depend for their existence on the practice of xenotransplantation. For a summary of the arguments, see Box 1.

Box 1 **Arguments about Xenotransplantation.**

**Recipient-based objections:** The organ may not fit well, be rejected and pass on zoonotic infections while the patient receives immunosuppressants. There may also be the moral or psychological cost of having killed the donor, perhaps in vain.
**Recipient-based response:** But customized, gene-edited, chimeric organs reduce the risks of rejection and physiological incompatibility, the need for immunosuppressants and zoonotic risks.
**Donor-based objections:** Yes, but reducing recipient-based objections by employing chimeras strengthens donor-based objections, as the donor is also now likely to suffer from harms produced by a chimeric condition, the use of cloning technology and the off-target effects of gene editing. And since chimeric donors will be genetically related to recipients, the moral costs for the participants of having harmed and killed a partly human relative may also increase.
**Response to donor-based objections:** A displacement of the cost and objections from recipient-based ones to donor-based ones is predictable when attempting to achieve improvements for humans at the expense of animals. However, all such objections are canceled at once by the realization that the donor has a life worth living and would not have existed had it not been a donor.

The following sections examine this last line of argument by drawing on the ongoing debate about what philosophers term ‘the Non-Identity Problem’. Section 1 discusses non-human potential donors and explains the role of transpigs: chimeras produced with a percentage of human genes and cells. Section 2 discusses the relevance of the Non-Identity Problem in three cases involving humans and three involving non-human animals. Section 3 explains some standard strategies to block a Non-Identity Defense of a case. It argues that although these strategies fail in the transpig case, appealing to the Non-Identity Problem does not suffice to cancel donor-based concerns. A final section reflects on other organ sources and two different futures for humanity.

## 2. The Choice of Donors

The enormous shortage of organs relative to patients in need of them has led to a search to increase the supply of donors and organs. More closely related donors may produce less tissue rejection but also sometimes prompt more serious moral objections. The worst-case scenario is illustrated by Michael Bay’s film *The Island*, which depicts a dystopia where self-aware human persons are raised in a colony with the promise of eventually traveling to an uncontaminated island. In fact, they are clones raised for others to harvest their organs should they be needed by the relevant client.

A version of *The Island* involving chimpanzees also seems objectionable. Chimpanzees are not human (because they have *pan troglodytes* rather than *homo sapiens* DNA), but they are considered persons because they satisfy the standard definition of personhood employed in metaphysics and bioethics, proposed by John Locke, as a ‘thinking intelligent being that has reason and reflection and can consider itself as itself, the same thinking thing, in different times and places’ [11], as well as other more demanding definitions proposed by other authors [12,13]. For example, chimpanzees pass the Gallup test for mirror self-recognition, can learn hundreds, sometimes thousands of words in sign language, and can even teach it, extending their collective vocabulary with compound concepts and metaphors. They can play complex games with rules, and display humor, curiosity, tool-making abilities, a theory of mind, interspecific altruism, and elaborate future plans [14,15]. Though the use of chimpanzees has diminished because of their scarcity and zoonotic risks, using non-human persons is arguably at least as morally objectionable as using human non-persons, such as irreversibly comatose humans with no more brain functions than those required for organ survival and no hint of consciousness.

Since using other primates involves further ethical and medical difficulties, ranging from their endangered status to blood type incompatibility, some scientists have settled on using a certain breed of pig. Pigs are tame and have organs of an appropriate size, and short and frequent pregnancies with large litters. Moreover, they are promising hosts for growing at least partly human organs by having some of the pig cells in their blastocysts replaced with human cells. Insofar as they grow from a blastocyst containing a mixture of pig cells and patient cells, they could be seen in some way as the patient’s ‘descendants’. Thus, while in ‘savior sibling’ cases, a mother gestates an additional child (whose alternative is not to exist) in order to save an existing child, in transpig cases, a patient uses a pig surrogate to gestate her transpig ‘descendant’ in order to save herself.

Unlike chimpanzees, pigs have not clearly passed the Gallup test for mirror self-recognition and are rarely if ever regarded as persons. Nevertheless, they are cognitively complex, highly social and emotional mammals, displaying curiosity and playfulness and an ability to learn dog-like tasks, such as herding or leading horses. They have individual personalities and experience strong emotions in anticipation of events and by perceiving the joy or gloom of others [16]. Given these characteristics, even if the balance of reasons does tip in favor of saving human lives through transpig xenotransplantation, it would be better if we could avoid using them to harvest organs by developing alternative techniques, such as cell printing or biodegradable scaffolds on which to grow organs.

Of course, it is true that large numbers of pigs are currently raised and slaughtered in far more horrific conditions than those conditions transpigs are likely to undergo, and for far more trivial culinary reasons than saving persons from death by organ failure. Some readers may therefore be puzzled by our concern for the welfare of donor transpigs. The absence of more demanding legal restrictions regarding pork production may well be relevant in assessing the legality, and perhaps also the morality of xenotransplantation using chimeric pigs. The ethical issues regarding transpigs, however, do not disappear merely by pointing to the existence of factory farming. These factory farms where pigs develop tumors and malformations, cannibalize each other, and die in large numbers before they reach the slaughterhouse should not exist, and so their current existence does not weaken the moral case for being concerned about the welfare of transpigs. It could be that both cases are morally problematic, and two wrongs do not make a right. Moreover, even if the case against factory farming is much stronger than the case against transpig xenotransplantation, there could be reasons to ban transpigs, even in the absence of a ban on factory farms. One such reason could be that perhaps only one such prohibition is feasible at a particular time. Another reason could be the existence of additional reasons that are not animal-centered (such as scientific uncertainty or risks to humans) but which count against using transpigs, although not against factory farms. In any case, even if eventually the balance of reasons favors allowing transpig xenotransplantation, the practice will still require regulations that take its morally problematic features into account. In short, the fact that factory farms are legally permitted does not directly establish that transpig xenotransplantation is morally permissible. For a summary of the arguments favoring the choice of pigs, see Box 2.

Box 2 **Why Pigs?**

**Morality.** The use of pigs is less morally objectionable than the use of chimpanzees because they are not self-aware persons like the very intelligent chimpanzee.
**Risks.** Chimpanzees are known to present serious zoonotic risks and pose additional risks to laboratory staff because of their strength and intelligence.
**Manageability.** Whilst chimpanzees are large, powerful, determined, hard to dominate, and good escape artists, pigs are tame, easy to control, and have more modest dietary needs.
**Abundance.** Pigs are not endangered, unlike so many primates. Instead, they are abundant and easy to breed in captivity, as they have short, frequent pregnancies and multiple litters.
**Compatibility.** Other species have organs of very different shapes and sizes, and have incompatible blood types.

The comparison with factory farms, however, may be used indirectly to argue for the permissibility of transpig xenotransplantation, as follows. Pigs reared to save human lives are likely to be kept in much better physical and emotional conditions, so that these pigs and their organs stay in optimal health. There will be no overfeeding, causing them fat liver disease in order to produce foie gras, and no killing them as piglets for their tenderness. Since these pigs are much smaller than humans, they will have to grow to full adult size, have a healthy weight, and suffer no pathologies, so that their internal organs will be fit for transplantation. As a result, they will enjoy longer lives in social groups with their relatives and will reach, we shall assume, the welfare threshold that makes the lives of pigs worth living, a level never reached in factory farms.

Under such conditions, one may argue that it is pigs that will be the first beneficiaries of xenotransplantation. This is not because the lives of pigs may one day be saved by such medical breakthroughs, but because *transpigs would have lives worth living and would otherwise not exist.* The next section explores a defense of the use of transpigs, constructed along similar lines.

## 3. Transpigs and the Non-Identity Problem

There is a common belief, amongst academics as well as the general public, that *for something to be morally bad, it has to be bad for someone*. This belief, which endorses what is sometimes termed ‘the person-affecting principle’, is challenged by what Derek Parfit has called ‘the Non-Identity Problem’ [17]. The Problem can be illustrated in diverse cases with one feature in common: an agent brings into existence some individuals, or groups, who have lives worth living that also contain various burdens that the agent could have avoided only by not bringing those specific individuals into existence. These cases generate a challenge because such actions lack *victims*, or specific individuals whose lives are made worse than they would otherwise be, but at least sometimes strike many of us as still wrongful. The Problem then is to explain what might make an action wrong even if it is victimless in the sense mentioned, or else to accept that there are no victimless wrongs and perhaps revise our judgments about particular cases accordingly.

For some well-known illustrations of the Problem, consider the following.

*Teenage Pregnancy*. There are many reasons to argue in favor of deferring motherhood to adulthood. Many such arguments appeal to the benefits of a mature mother for the potential son or daughter. If the mother waits, however, she may then change partners and she will certainly change eggs, and so the alternative history for a potential child of a non-adult is not to be born at all. Thus we cannot invoke the specific child’s improved welfare as an argument against conception, even in the case of very young teen mothers.

*Wrongful Life*. Adult mothers may be wrongly advised by their doctors not to delay their pregnancy until the effects of some medication or illness fully subside. They may thereby conceive a child with a disability whose other option, again, was not to be born. Providing the resulting life is worth living, the individual enjoying it cannot complain that the mistaken advice made them worse off than they would have otherwise been, nor can the mother who loves and loves to have *this* child. Some parents have also deliberately decided to conceive a child with a disability, like deafness, when they could have had a hearing, but also different, child.

*Risky Policy*. In the face of climate change, a society might evolve by maintaining a population dependent on renewable energy, or by employing nuclear energy. Suppose that because individuals meet different partners in these different scenarios, and so conceive different offspring, the society’s population is composed of entirely different individuals after a sufficiently long period. Suppose too that although every member of society has a life worth living, the nuclear population suffers various radiation-related pathologies and its members are unlikely to survive beyond 45. Such a variation in future members’ quality of life would normally favor choosing social policies that produce the green rather than the nuclear population, but the fact remains that none of the individuals composing the nuclear population have been made worse off than they would have been made by choosing greener policies. For a summary of the Non-Identity Problem, see Box 3.

Box 3 **The Non-Identity Problem.**

One familiar way to explain what makes a choice wrongful appeals to the way in which the choice makes other beings worse off than they would have been had the agent chosen differently. This type of moral argument, which claims that a choice is wrong because it has *victims* who are *harmed* compared with how they might otherwise have fared, is inapplicable in cases where we make *identity-determining choices* that affect not only the quality of life of other beings, but also which specific beings will exist.

For example, suppose that if a couple chooses to conceive immediately then, due to some temporary medical condition, they will create a disabled child who has a life worth living but also a serious welfare-diminishing condition. The couple could, however, choose to defer conception and create a different child with no comparable disability and a higher quality of life. Reflecting about such choices, many are convinced that immediate conception might at least sometimes be wrong, even if the choice is not worse for any specific individual including the disabled child, since the couple could not have chosen to create her without her disability.

If we accept this type of conclusion then we face the Non-Identity Problem, or the challenge of explaining what might make choices wrongful *even when they lack victims*. If no plausible answer exists and there is no harmless wrongdoing, then many of us need to revise our convictions about which choices are wrongful.

When faced with these types of case, some respondents think the absence of specific victims makes a major difference to how the concrete cases should be assessed. They conclude that the outcomes produced by teenage pregnancy, medical misdiagnosis, and the choice of the nuclear policy are not bad, or at least not as bad as they first appear. Others argue, like Parfit himself in *Reasons and Persons,* that the absence of specific victims makes no difference, and instead conclude that these cases challenge the general principle that ‘for something to be *bad* it must be *bad for* someone’. Since these cases, and indeed most of the cases discussed in the literature on the Non-Identity Problem, involve human persons, this belief is known as the *person*-affecting principle. This is a misnomer, however, because the principle is not essentially related to persons. Some have re-named it the ‘individual affecting principle’ [18] or the ‘identity-affecting principle’ [19], because ‘someone’ could refer to any rigidly designated individual, regardless of whether it is a human (or chimpanzee) person, a comatose human nonperson, or a nonhuman nonperson, like a hen or a fish [20]. The third case is relatively neglected in the non-identity literature [21,22,23,24], but here are some relevant cases that are not person-involving. Perhaps the most famous case of animal non-identity is that of Dolly the Sheep, assuming her short, disease-ridden life was worthwhile. Other cases include the following.

*Designer Dogs*. People have always selectively bred dogs that were, for example, good shepherds, but there is now an endless whim list that dog breeders try to satisfy. Dogs must be hypoallergenic, with minimal walking and eating needs, must not bark or shed hair, must fit into the travel bags of budget airlines and must massage their owners’ egos with utter dependence on their affection, even starving themselves in the owner’s absence. There is even some demand for tiny dogs that die young just as children tire of them. Intense trait selection through forced inbreeding leads to dogs with chronic pain, and numerous disabilities and health problems, whose other option, however, is not to exist.

*Oncomice.* This transgenic mouse can carry cloned genes integrated into its own genome that will cause the mouse to develop cancer early in life, so that it can be subjected to different therapies before it is killed to study the impact of such therapies on its tumors. Housed in a stable group with other familiar mice, protected from thirst, hunger, cold, heat, or predators, we assume they will meet a welfare threshold for worthwhile mouse lives.

*Transpigs.* As an aging population increases the demand for organ transplants, partly human pigs grow organs in comfortable facilities, emotionally and physically appropriate for their development. We assume they will meet the welfare threshold for worthwhile pig lives.

One may think that the case of transpigs is comparable to that of other animals, such as the mice used in so many tests. However, even leaving the cognitive differences between pigs and mice aside, mice, which are already naturally short-lived, already exist abundantly in nature and so their existence does not depend on a medical procedure. Transpigs, by contrast, are human creations, like Dolly, genetically engineered with cloning technology to be a certain kind of pig combined with human DNA, and designed to concentrate more human cells in certain organs. Transpigs, moreover, are heavily edited with CRISPR to eliminate immune-provoking sugars from the surface of pig cells, to introduce human genes that regulate blood coagulation and clot formation, and to excise viral sequences which could press zoonotic risks. Finally, transpigs will be engineered to save a particular patient whose DNA they will carry. So one may argue that *each* particular genetically engineered, CRISPR edited, chimeric transpig owes its existence to the procedure for which it has been created. Like other chimeras, they are likely to die young and suffer complications derived from their chimeric condition, which may also explain the high rate of chimeric fetal deaths [25]. Complications are also likely to increase with increases in the human percentage of the chimera to reduce immunological rejection and physiological incompatibility, and so transpigs can be considered, like short-lived dogs and oncomice, doomed from the start.

One may still argue, however, that whatever built-in deficiencies transpigs will carry, their exact time of death will eventually depend on the need for their organs, and so will depend, unlike the deaths of oncomice and short-lived dogs, on human choices that impose avoidable harms on them. This difference is not so clear, however, because the exact time of death of oncomice and short-lived dogs is also contingent and dependent on human actions, rather than entirely determined by the creature’s identity. Oncomice will eventually develop cancer, but the time at which this will happen will be affected by various conditions under examination. In addition, the suffering and eventual death of an oncomouse will also depend on the chemotherapy or radiotherapy it endures, and so neither the suffering nor the time of death is entirely invariant regardless of human action. The case of Designer Dogs is even clearer as some owners will give their dogs greater medical attention than others, and even their lifestyle will greatly alter how long they will live until, for example, their owners decide on euthanasia. Thus, the three animal cases of non-identity are not that different after all. It is unclear, moreover, that whatever difference remains between these cases and that of transpigs makes the arguments applicable to oncomice and short-lived dogs inapplicable to transpigs.

Much the same happens with the human cases. Disability activists insist their suffering and even their disability is entirely contingent. Hence the slogan: ‘the problem is not the wheelchair but the staircase into the building’. The case of teenage motherhood is even clearer. In certain societies it need not be a disadvantage at all. In fact, even in the imaginary, highly idealized case of Risky Policy, the suffering endured by the nuclear generation and their exact time of death will also be contingent and dependent on factors such as the existence of a high-quality public health care system.

In the case of transpigs, even those with a very small percentage of human cells have a major inbuilt tendency to premature death and will endure additional life-shortening challenges, such as being immuno-compromised due to cloning technology, and being heavily CRISPR-edited to reduce zoonotic risks and histological rejection. As a result, transpigs are even more likely to be short-lived than some designer dogs. In addition, for different medical reasons, transpigs might be designed with additional kinds of built-in obsolescence, so to speak, essential to their lives. For example, given the larger size of human beings, transpigs could grow human organs that continue to increase in size, eventually killing the transpig if the organs are not removed. Or transpigs could be designed to survive with certain doses of immunity suppressants but die of an immune-related condition the moment the suppressants are canceled or reduced, much like oncomice will die without any cancer treatment. Finally, given that small, short-lived dogs already exist, scientists could also design small, short-lived transpigs with small organs appropriate for transplantation to human infants. In sum, the case of transpigs is likely to resemble that of oncomice or designer dogs not only because transpigs can be deliberately made short-lived, but also because they are likely to be short-lived and suffer from other difficulties as a by-product of having been heavily edited and designed with certain chimeric traits. They will certainly resemble oncomice and short-lived dogs more than natural species.

If so, it could be argued that, providing their lives are sufficiently good, the practice of transpig xenotransplantation will not make specific transpigs worse off than they might otherwise have been, and that *this fact undermines harm-focused donor-based objections to chimera xenotransplantation*. The next section explores various ways of challenging the appeal to the Non-Identity Problem to undermine donor-based concerns with chimeric xenotransplantation. For a list of human and non human cases of non-identity, see Box 4.

Box 4 **Non-Identity Cases.**

**Human**

**Animal**
Teenage Pregnancy    OncomiceWrongful LifeDesigner DogsDeaf ParentsChimerasRisky PolicyDolly the Sheep

## 4. Can the Fact of Non-Identity Undermine Donor-Based Concerns?

This section examines standard responses to the Non-Identity Problem that are inapplicable or face difficulties in the case of transpigs, a case which appears to be particularly intractable. Despite such difficulties, we eventually resist the claim that the Problem undermines donor-based objections to the use of transpigs.

*The Virtue Ethics Challenge*. Discussing the Non-Identity Problem, Claire Palmer appeals to virtue ethics to criticize the attitudes of people who rush into parenthood or who order the production of inbred, unhealthy, or short-lived dogs. Their attitude, she argues, ‘implies an unwillingness to accept the inconvenience, the emotional commitment and the long-term caring for another sentient being that is appropriate’ [24]. It thus ‘reflects the absence of human traits that we admire and regard as morally important’ [26].

Palmer admits that the people she criticizes may reply by denying their behavior was unvirtuous, given that the human offspring’s or dog’s only other option was not to exist. So, although Palmer deems this the best solution to the Non-Identity Defense of short-lived dogs, she admits that it is not foolproof. Moreover, we may deem unvirtuous the insufficiently concerned attitudes of the risky policy enforcers, the negligent doctor, the deaf parents, the impatient mothers or the owners of the unhealthy or short-lived dogs, because they all seem to act impatiently, and without good reason. Transpig breeders, however, attempt to save human lives, and so we would not normally describe them as acting on a whim or behaving impatiently.

*The Placeholder Challenge*. Some argue that the above assumption that chimeric xenotransplantation is victimless and thus morally unproblematic, at least from a donor-based perspective, can be challenged as follows. Suppose that a couple stipulate in their will that their fourth child should inherit their car [27]. The child may not have even been conceived at the time. But this is irrelevant to the testament: whoever comes fourth, gets the car. We can extend this to the discussion of future generations and establish, for example, that they should inherit a certain amount of natural resources and avoid radioactive waste. We could also believe that whatever companion dog we came to have should have a normal life-span. Extending this reasoning to the case of transpigs is more complicated. First, while any dog may do, transpigs are individually designed for a patient, and so are not equally replaceable tokens of a certain kind. Moreover, while future generations may inherit very different environments, with transpigs there is no similar leeway, if they are to remain capable of producing the healthiest organs.

*The Species-Norm Challenge.* Another way to respond to the assumption, which is often employed in debates about human enhancement, involves appealing to species normality: abstaining from producing the best possible child is not to harm the child because harm must be defined by reference to the species norm, for example, in terms of ‘pathology’ [28]. Clearly, death by 45 and having multiple radioactivity-related difficulties are pathologies, so we can explain opposition to the Risky Policy in this way.

Appealing to species normality, however, makes less sense when a creature does not belong solely to a single species. In fact, the idea of ‘chimeric normality’ seems oxymoronic. Assuming, however, that transpigs could have their own trans-species normality, it is not clear that the lives of transpigs, pampered like savior children, are worse than those experienced by pigs in the wild. Life in nature is not always idyllic [29,30]. Like the ‘bubble-babies’ suffering from combined immunodeficiency, transpigs will have their freedom limited in order to protect them from disease. This would be a very serious objection to the use of transchimpanzees. In the case of transpigs, however, at least some animal advocates believe that, providing they have ample and comfortable facilities, sufficient freedom of movement, and association with others, the objection may not be too strong [31]. The reason is that unfreedom is bad for nonpersons only when it translates into welfare losses. Imagine, for example, a school of fish that cannot distinguish the pond where they live from the pond where they are trapped, nor plan to escape or imagine themselves elsewhere. These creatures have a right not to suffer in the pond, but not to be free from it. In contrast, persons have an interest in freedom or non-interference with their decisions that outweighs their interest in comfort, protection or longevity. But not all creatures, human infants included, have a comparable interest in non-interference for its own sake. Thus, neither the species norm nor the associated freedom objection can easily apply to transpigs.

*The Consequentialist Challenge.* The nineteenth century British philosopher Leslie Stephen observed: ‘The pig has a stronger interest than anyone in the demand for bacon. If all the world were Jewish, there would be no pigs at all’ [32]. Peter Singer responds to Stephen’s observation by appealing to the bad consequences of pig farming, including all the human lives a vegetarian diet could save and the suffering involved in factory farming, which eliminates any interest pigs could have in being alive under such conditions [33]. Singer’s response, however, is inapplicable to the case of transpigs, which are better candidates for a Non-Identity Defense than normal pigs, as the latter could exist outside a bacon-producing program. Transpigs, we have assumed, have lives worth living, and they are used to save human beings from organ failure. Thus, whilst one may appeal to consequentialism to reject other non-identity cases like Risky Policy, Teenage Pregnancy, or Wrongful Life, it is unclear that consequentialism condemns transpigs.

*The Non-Comparative Harm Challenge.* We normally think that an individual has been harmed by an action when she has been made *worse off than she would otherwise have been* had that action not been performed. But some philosophers advocate a non-comparative view of harm, proposing instead that ‘one harms someone if one causes him pain, physical or mental discomfort, disease, deformity, disability, death’ [34], irrespective of comparisons with alternative courses of history including one involving the individual’s non-existence [35]. This view could explain why we think the ‘victims’ of Risky Policy, Teenage Pregnancy, and Designer Dogs have been harmed, even when they cannot exist in a better state. Some find this view problematic because it involves appealing to rights that *could not be fulfilled*, for example, because it was impossible for the people resulting from premature pregnancies to be born at that point and yet avoid certain harms [17].

In the transpig case, if the life is worth living, and short, but not bad, as we have so far assumed, the main harm will be the killing. But here two complications emerge. First, it is difficult to explain how non-persons, who are not future-oriented nor psychologically contiguous with their future self, are harmed by receiving a decent life that ends with a painless death. For example, if a prawn is not more connected to its future self than it is connected to another prawn, then it is unclear that it is bad if one prawn dies painlessly and is replaced by another. Second, as Luhan Yang, one of the main scientists involved in xenotransplantation has argued, even if we grant that the transpig is harmed by its death, the alternative is for eight human persons to be harmed by their deaths [36,37]. Since few would condemn a swineherd who kills one pig to save another eight pigs from infection, transpig xenotransplantation seems like the harm-minimizing option, and so appealing to non-comparative harm is less effective here than in other cases, like Risky Policy.

There is a difference, however, between claiming that using transpigs is the harm-minimizing option, or that the appeal to non-comparative harm faces some difficulties in this case, and saying that the Non-Identity Problem can entirely undermine any donor-based concerns. Even if harms to transpigs are eventually, all things considered, deemed justified, they remain a pro-tanto reason against the practice. This would be easier to see if the victims of an organ-harvesting scheme were human persons who had been bred and designed for that purpose. In such a case, most of us would not hesitate to condemn the treatment, both because of the harms it produced and because those persons were being used rather than merely harmed, in a way that was foreseeable and avoidable. And even if we grant that transpigs have less to lose from losing their lives, and that they owe those lives to xenotransplantation, we cannot conclude that the Non-Identity Defense suffices to cancel all concerns with harms to chimeric donors. Finally, as one reviewer noted, there can be moral objections to xenotransplantation that are not harm-based [38]. Thus, we may grant that transpigs are not actually harmed by xenotransplantation because their lives are worthwhile, and their only other option is not to exist. We may even deny that transpigs suffer any actual or potential gross or net harms, but since there can be moral objections that do not appeal to harm, we cannot yet conclude that donor-based objections have thereby entirely withered away. For a summary of the peculiarities of the transpig case, see Box 5.

Box 5 **Features of the Transpig Case that Make Replying to the Non-Identity Problem Particularly Difficult.**

- Mothers that would not wait, policy makers who produce the nuclear population, and pet owners who commission designer dogs display insufficient concern with suffering, or the wrong attitude for the responsibility they are acquiring. Lack of concern, however, does not explain transpig breeders’ behavior.
- Whilst any mouse or dog can be chosen, specific chimeras are created for a patient.
- Defining harm relative to species normality is difficult when two species are involved.
- Although dogs and mice can suffer more or less depending on how we treat them, there is little leeway regarding how transpigs must be treated.
- Whilst designer dogs may produce trivial benefits, and oncomice may or may not produce major benefits, transpigs will save human persons.
- Whilst designer dogs and oncomice suffer ill-health and disability, the main harm for transpigs may be premature death, but the badness of death is particularly hard to explain in non-persons.
- If using a transpig can save eight human persons, then xenotransplantation minimizes harm.

## 5. Concluding Remarks: The Relevance of Alternatives

So far, we have resisted the claim that the Non-Identity Defense undermines donor-based concerns. In concluding, we will question the way the debate over xenotransplantation often proceeds, and draw attention to some important recipient-based concerns.

Advocates of xenotransplantation often proceed by balancing the life that a transpig owes to xenotransplantation against the lives of a larger number of human persons. Others more critical of xenotransplantation focus on the practice more generally. They attend to the risk to recipients as well as the harms to donors it generates, and contrast the practice with some alternative practice, like cell-printing or scaffolding, that reduces those risks and harms. When evaluating the case for a moratorium on the use of transpigs, we should not only weigh the certain harms and their possible justifications in terms of non-identity, self-defense, or human need, as we have done here so far. In addition, we need to adopt a more general perspective, and take into account at least the following factors.

First, we must attend to risks not only to donors and recipients but also to *third parties*, whether human or not, who may face, for example, zoonotic risks. Second, we need to focus on xenotransplantation procedures not only in their perfected form but also consider the moral and non-moral costs involved in their *development*, including the large number of discarded transpigs and failed transpig transplants that will take place as the procedure is refined. Third, we must not assume that procedures will always take place in conditions of *perfect compliance* with the relevant rules and protocols but assume human failure and human misconduct will sometimes take place. Fourth, we must consider the practice not only within a single research center or even a single country [39].

For example, we may feel confident that our own governments will prohibit transpig xenotransplantation until the technique is perfected and will never permit the use of pigs that have not been properly edited, thus risking zoonotic contamination. Nevertheless, we cannot be certain how all governments will act. *Xenotourism*, the practice of traveling to another country to acquire an organ unavailable where a patient resides, could make it impossible for any country to protect itself from zoonotic risks. An individual returning from abroad with a partly non-human organ that was not properly prepared could eventually infect not only friends and relatives but also sanitary staff. Given that an illicit market in human organs is one of the arguments voiced in favor of transpig xenotransplantation, we should not lose sight of the fact that an illicit market in transpig organs may also emerge [9]. After all, we know that there is a race to be the first scientist to perform certain procedures, and that this does not depend only on scientific genius, technological sophistication, or funding, but also on the ethical committees and protocols that can stop or delay research. It would thus be unsurprising if countries with less strict protocols attracted more xenotourists.

One might reply to the previous argument by claiming that it in fact supports allowing chimeric xenotransplantation in countries with relatively strict regulations. For nothing will promote xenotourism in loosely regulated countries more than a complete prohibition on xenotransplantation in more strictly regulated countries. However, allowing xenotransplantation in one country will not suffice to stop its patients from traveling abroad to obtain chimeric organs either. There may be patients who do not qualify for the procedure where they reside, patients who are concerned with anonymity, patients seeking a cheaper price, and so on. Moreover, whether something is allowed or not within one country sends a signal to its citizens about both safety and moral permissibility which could increase these citizens’ attitudes to taking risks abroad. So, the risks remain real. By contrast, if alternative organ-producing methods are developed, there may not be any need for xenotransplantation either domestically or abroad.

Fifth, we need to examine the range of feasible *alternatives* to xenotransplantation as a means to increase the supply of organs. These include not only alternative scientific procedures, like cell printing or biodegradable scaffolding [40], but also legal measures, such as: the presumption of consent to be a posthumous donor in the absence of contrary evidence; the reduction of relatives’ veto powers over donations; enhancing priority in access to organ transplants to individuals willing to make their organs available after their deaths; and securing greater international cooperation to find suitable matches. With an aging population, such measures may not suffice but could still (i) buy us some time to perfect safer procedures, and (ii) alter the permissibility of producing organs via xenotransplantation. For there is a major moral difference between imposing certain risks to others before, or after, alternative solutions have been exhausted. To be sure, it is not up to individual scientists, like Yang, to determine donor national policies nor to secure greater international cooperation. Instead all of us have a responsibility to press politicians to address these issues rather than leave scientists to solve the scarcity problem alone and then criticize them when things go wrong.

Finally, we need to look at what sort of future for humanity we are influencing when we support one procedure rather than another. We appear to be at a fork in the road leading to two different futures for us, and for other species too. One is the aseptic future in which we no longer rear animals to eat or to harvest their partly human organs. Thanks to cell-growing techniques, our laboratories will be able to grow uncontaminated, antibiotic- and infection-free ‘clean meat’ [41], and any organs that we or our companion animals may need. Another is the future in which we ‘colonize’ other species, modifying or even fusing ourselves with them, for example, by producing partly human animals to act as organ banks, and perhaps even incorporating into ourselves not only those partly non-human organs, but also genes from other species.

The difference between extraction and immersion futures is momentous. In one, we extricate ourselves from other species, growing tissues in sterile lab containers; in the other, we intertwine ourselves with other species, adapting them to our purposes, perhaps taking from one species the means to immunize ourselves from problems that mixing ourselves with another species has created. There should be greater public awareness and debate about the choice between the extraction and immersion strategies. Faced with an ‘all or nothing’ decision, from a collective and global perspective, extrication seems the more prudent and less morally problematic option, even if we cannot blame individual patients who prefer queuing in whatever is the fastest line.

## References

[1] Rodríguez-Arias D., Smith M.J., Lazar N.M. (2011). Donation after Circulatory Death: Burying the Dead Donor Rule. Am. J. Bioeth..

[2] Rodríguez-Arias D., Ortega-Deballon I. (2012). Protocols for Uncontrolled Donation after Circulatory Death. Lancet.

[3] Wilkinson T.M. (2007). Racist Organ Donors and Saving Lives. Bioethics.

[4] Davies N.K. (2018). Woman Gives Birth Using Womb Transplanted from Dead Donor. The Guardian.

[5] Savulescu J., Beauchamp T.L., Frey R.G. (2012). Genetically Modified Animals: Should There Be Limits to Engineering the Animal Kingdom?. Oxford Handbook of Animal Ethics.

[6] Scutti S. (2017). First Human-Pig Embryos Made, Then Destroyed. CNN.

[7] He Jianku Defends ‘World’s First Gene-Edited Babies’. https://www.bbc.com/news/world-asia-china-46368731.

[8] Lonbardo C. http://visionlaunch.com/pros-and-cons-of-xenotransplantation.

[9] Lombardo C.R. Eight Crucial Pros and Cons of Xenotransplantation. https://connectusfund.org/8-crucial-pros-and-cons-of-xenotransplantation.

[10] Lonbardo C. https://www.sciencedaily.com/releases/2013/07/130731093255.htm.

[11] Locke J. (1983). Essay Concerning Human Understanding.

[12] Fletcher J. (1972). Indicators of Humanhood. Hastings Cent. Rep..

[13] Jaworska A. (2007). Caring and Full Moral Standing. Ethics.

[14] Cavalieri P., Singer P. (1993). The Great Ape Project.

[15] Fouts R. (1997). Next of Kin.

[16] Marino L., Colvin C. (2015). Thinking Pigs. Int. J. Comp. Psychol..

[17] Parfit D. (1984). Reasons and Persons.

[18] McMahan J., Roberts M., Wasserman D. (2009). Asymmetries in the Morality of Causing People to Exist. Harming Future People: Ethics, Genetics Non-Identity Problem.

[19] Lillehammer H. (2009). Reproduction, Partiality and the Non-Identity Problem. Harming Future People.

[20] DeGrazia D. (2005). Human Identity and Bioethics.

[21] Rollin B. (2003). Farm Animal Welfare: Social, Bioethical and Research Issues.

[22] Sandøe P., Christiansen S. (2008). Ethics of Animal Use.

[23] Streiffer R. (2008). Animal Biotechnology and the Non-Identity Problem. Am. J. Bioeth..

[24] Palmer C. (2012). Does Breeding a Bulldog Harm it? Breeding, Ethics and Harms to Animals. Univ. Fed. Anim. Welf..

[25] Wu J., Platero-Luengo A., Sakurai M., Sugawara A., Gil M.A., Yamauchi T., Suzuki K., Bogliotti Y.S., Cuello C., Valencia M.M. (2017). Interspecies Chimerism with Mammalian Pluripotent Stem Cells. Cell.

[26] Hill T., LaFollette H. (2007). Ideals of Human Excellence and Preserving Natural Environments. Ethics in Practice.

[27] Wolf C. (2009). Do Future Persons Presently Have Alternate Possible Identities?. Harming Future People.

[28] Harman E. (2004). Can We Harm and Benefit in Creating?. Philos. Perspect..

[29] Faria C. (2016). Why We Should Not Postpone Awareness of Wild Animal Suffering. Anim. Sentience.

[30] Horta O. (2010). Debunking the Idyllic View of Natural Processes: Population Dynamics and Suffering in the Wild. Telos.

[31] Cochrane A. (2012). Animal Rights Without Liberation.

[32] Singer P. (1979). Practical Ethics.

[33] Singer P. (1979). Practical Ethics.

[34] Harman E. (2009). Harming as Causing Harm. Harming Future Persons.

[35] Holtug N. (2001). On the Value of Coming into Existence. J. Ethics.

[36] Yang L. How to Create a World Where No One Dies Waiting for a Transplant. https://www.ted.com/talks/luhan_yang_how_to_create_a_world_where_no_one_dies_waiting_for_a_transplant#t-785440.

[37] Harris J. (1975). The Survival Lottery. Philosophy.

[38] Feinberg J. (1990). Harmless Wrongdoing: The Moral Limits of the Criminal Law.

[39] Mohiuddin M. (2007). Clinical Xenotransplantation of Organs: Why Aren’t We There Yet?. PLoS Med..

[40] Minorov V., Boland T., Trusk T., Forgacs G., Markwald R.R. (2003). Organ Printing: Computer-Aided Jet-Based 3D Tissue Engineering. Trends Biotechnol..

[41] The Good Food Institute The Future of Meat. http://cleanmeat.org/.

